# Does Antenatal Betamethasone Alter White Matter Brain Development in Growth Restricted Fetal Sheep?

**DOI:** 10.3389/fncel.2020.00100

**Published:** 2020-04-30

**Authors:** Amy E. Sutherland, Tamara Yawno, Margie Castillo-Melendez, Beth J. Allison, Atul Malhotra, Graeme R. Polglase, Leo J. Cooper, Graham Jenkin, Suzanne L. Miller

**Affiliations:** ^1^Department of Obstetrics and Gynaecology, The Ritchie Centre, Hudson Institute of Medical Research, Monash University, Clayton, VIC, Australia; ^2^Department of Paediatrics, Monash University, Clayton, VIC, Australia

**Keywords:** fetal growth restriction, FGR, IUGR, glucocorticoids, brain injury, neuropathology, preterm

## Abstract

Fetal growth restriction (FGR) is a common complication of pregnancy often associated with neurological impairments. Currently, there is no treatment for FGR, hence it is likely these babies will be delivered prematurely, thus being exposed to antenatal glucocorticoids. While there is no doubt that antenatal glucocorticoids reduce neonatal mortality and morbidities, their effects on the fetal brain, particularly in FGR babies, are less well recognized. We investigated the effects of both short- and long-term exposure to antenatal betamethasone treatment in both FGR and appropriately grown fetal sheep brains. Surgery was performed on pregnant Border-Leicester Merino crossbred ewes at 105–110 days gestation (term ~150 days) to induce FGR by single umbilical artery ligation (SUAL) or sham surgery. Ewes were then treated with a clinical dose of betamethasone (11.4 mg intramuscularly) or saline at 113 and 114 days gestation. Animals were euthanized at 115 days (48 h following the initial betamethasone administration) or 125 days (10 days following the initial dose of betamethasone) and fetal brains collected for analysis. FGR fetuses were significantly smaller than controls (115 days: 1.68 ± 0.11 kg vs. 1.99 ± 0.11 kg, 125 days: 2.70 ± 0.15 kg vs. 3.31 ± 0.20 kg, *P* < 0.001) and betamethasone treatment reduced body weight in both control (115 days: 1.64 ± 0.10 kg, 125 days: 2.53 ± 0.10 kg) and FGR fetuses (115 days: 1.41 ± 0.10 kg, 125 days: 2.16 ± 0.17 kg, *P* < 0.001). Brain: body weight ratios were significantly increased with FGR (*P* < 0.001) and betamethasone treatment (*P* = 0.002). Within the fetal brain, FGR reduced CNPase-positive myelin staining in the subcortical white matter (SCWM; *P* = 0.01) and corpus callosum (CC; *P* = 0.01), increased GFAP staining in the SCWM (*P* = 0.02) and reduced the number of Olig2 cells in the periventricular white matter (PVWM; *P* = 0.04). Betamethasone treatment significantly increased CNPase staining in the external capsule (EC; *P* = 0.02), reduced GFAP staining in the CC (*P* = 0.03) and increased Olig2 staining in the SCWM (*P* = 0.04). Here we show that FGR has progressive adverse effects on the fetal brain, particularly within the white matter. Betamethasone exacerbated growth restriction in the FGR offspring, but betamethasone did not worsen white matter brain injury.

## Introduction

Fetal growth restriction (FGR) has many etiologies but most commonly occurs as a result of placental dysfunction, and is a major cause of perinatal mortality and, in survivors, morbidities related to pulmonary, cardiovascular and neurological structure and function (Malhotra et al., 2019). The presence of placental dysfunction is a defining feature of FGR (Gordijn et al., 2016), resulting in a reduction in the amount of oxygen and nutrients reaching the fetus, thus reducing fetal growth. As a result, the growth-restricted fetus is hypoxic, hypoglycaemic and hypercortisolemic (McMillen et al., 2001; Lipsett et al., 2006) and it is these states that are thought to contribute to the increased morbidities associated with FGR. The fetus mounts an adaptive response to chronic hypoxia (Giussani, 2016), which involves altered cardiovascular output to preferentially spare vital organ development in the brain and heart, called *brain sparing*. This term is however a misnomer and brain sparing does not guarantee normal development in the growth-restricted fetus (Miller et al., 2016). FGR is strongly associated with developmental deficits in brain structure and function, with long-term sequelae including reduced motor skills, memory and cognition, and altered neuropsychological behaviors (Miller et al., 2016). The most significant neurological problems are linked to the most severely affected growth-restricted infants who are born preterm (Schreuder et al., 2002; Baschat, 2014).

Infants at risk of preterm birth before 34 weeks gestation will be exposed to antenatal glucocorticoids *via* maternal administration to improve neonatal survival (Crowley, 2000). The beneficial effects of antenatal glucocorticoids—either betamethasone or dexamethasone—are well proven; a single course of antenatal glucocorticoids administered to at-risk pregnancies <34 weeks increases infant survival by 50% and decreases the rate of respiratory distress syndrome (RDS) by about the same degree (Crowley, 2000). Glucocorticoids promote lung maturation by increasing the production of surfactant, promoting lung structural maturation and enhancing the clearance of lung liquid (Liggins, 1994; Wallace et al., 1995). At the cellular level, endogenous and exogenous glucocorticoids mediate organ maturation *via* regulation of cell proliferation, differentiation, and apoptosis, and glucocorticoids are powerful mediators of vascular function (Fowden et al., 1998; Yang and Zhang, 2004; Michael and Papageorghiou, 2008). These cellular effects are critical in the lung to promote neonatal survival after preterm birth, but antenatal glucocorticoids also act on the developing brain. Exogenous glucocorticoids increase cerebral vascular resistance leading to decreased cerebral blood flow (Schwab et al., 2000; Miller et al., 2007) and impair cerebral oxygen delivery in a region-specific manner (Schwab et al., 2000). These changes in cerebral blood flow are associated with altered electrocortical activity, suggestive of dysfunctional complex neuronal activity and disturbed cerebral metabolism (Schwab et al., 2001). Dexamethasone, in particular, induces acute EEG hyperexcitability and sustained alterations in ovine fetal sleep patterns (Davidson et al., 2011). At the cellular level, synthetic glucocorticoids disrupt myelination within the brain of appropriately grown fetal sheep (Antonow-Schlorke et al., 2009), and reduce the neuronal number in fetal primates (Uno et al., 1990).

While multiple studies have examined the effects of antenatal glucocorticoids on the brain of appropriately grown (and otherwise healthy) fetuses, the effects of glucocorticoids on the developing FGR brain are less documented. In the absence of exogenous glucocorticoid exposure, we have shown significant neuropathology in growth-restricted fetal sheep and newborn lambs that includes white matter hypomyelination, axonal injury, neuroinflammation, increased cellular apoptosis, and altered vascularization (Miller et al., 2014; Castillo-Melendez et al., 2015; Alves de Alencar Rocha et al., 2017). The hemodynamic response of the FGR fetus is markedly different from that of the appropriately grown fetus (Schwab et al., 2000; Miller et al., 2007), mimicking differences seen between FGR and normally grown human fetuses (Wallace and Baker, 1999). The appropriately grown fetus responds to betamethasone with vasoconstriction, decreased cardiac output and decreased cerebral blood flow (Miller et al., 2009). In contrast, the FGR fetus responds with widespread systemic vasodilatation, increased cardiac output and increased blood flow to all major organs including the brain (Miller et al., 2009). Following betamethasone, cerebral blood flow in the FGR fetus shows a biphasic response, with an initial vasoconstriction followed by a prolonged increase in brain blood flow (Miller et al., 2007). This closely mimics what is seen clinically where betamethasone causes increased placental and cerebral blood flow in the FGR fetus (Wallace and Baker, 1999; Edwards et al., 2002), indicative of systemic vasodilatation. It remains unknown whether these differential hemodynamic responses in the FGR vs. appropriately grown fetus have an exacerbating effect on neuropathology.

Therefore, in the current study, we examined whether antenatal betamethasone induced brain injury in growth-restricted fetal sheep and if neuropathology was exacerbated in FGR compared to appropriately grown fetuses. We collected brains for histological analysis both immediately following glucocorticoid exposure and at 10 days following glucocorticoid administration to determine whether longer exposure to antenatal glucocorticoids would further impact neuropathology. We hypothesized that exposure to antenatal betamethasone would induce a greater degree of white matter brain injury in FGR fetuses compared to appropriately grown fetuses, and that this would remain evident 10 days later.

## Materials and Methods

Experimental procedures were approved by the Monash Medical Centre Animal Ethics Committee A (MMCA2010/23, MMCA2011/39), and complied with the National Health and Medical Research Council Australia Code of Practice for the Care and Use of Animals for Scientific Purposes.

Surgery was performed on 34 singleton- or twin-bearing Border-Leicester Merino crossbred ewes at 105–110 days gestation (term 150 days). Ewes in the 125-day cohort were given medroxyprogesterone acetate (MPA; 300 mg intramuscularly; i.m.; Pfizer, Australia) 1 day before surgery to prevent preterm labor in response to glucocorticoid exposure (Jenkin et al., 1985). On the day of surgery, all ewes received an intravenous (i.v.) dose of ampicillin (Austrapen, 1g, CSL, Limited, Australia) before the induction of anesthesia with sodium thiopentone (Pentothal, 20 mg/kg, i.v.; Bomac Laboratories Limited, New Zealand). General anesthesia was maintained with 2.5% isoflurane (Isoflo, Abbott Private Limited, Australia) in oxygen and nitric oxide (70:30). Each fetus was exteriorized and the umbilical cord exposed before a small incision was made in the sheath surrounding the umbilical cord, approximately 3 cm from the fetal abdomen, to allow for single umbilical artery ligation (SUAL) to induce FGR. We have previously used this model to successfully induce FGR in sheep (Miller et al., 2007, 2012, 2014). Control fetuses had their cord manipulated but not ligated. All fetuses were implanted with a femoral artery catheter [inner diameter (ID) 0.8 mm, outer diameter (OD) 1.5 mm, Dural Plastics, Australia] and a catheter in the amniotic sac (ID 1.5 mm, OD 2.7 mm, Dural Plastics, Australia). Ewes received a jugular vein catheter (ID 1.5 mm, OD 2.7 mm, Dural Plastics, Australia) for the administration of antibiotics.

For 3 days following surgery, ampicillin was given to the ewe (500 mg i.v.) and into the fetal amniotic sac (500 mg into each amniotic catheter) and a daily fetal blood sample was taken for analysis of blood gas parameters (ABL700 blood gas analyzer, Radiometer, Denmark) to monitor fetal wellbeing. Ewes were then treated with a clinical dose of betamethasone (11.4 mg i.m.; Celestone Chronodose, Schering Plough, Australia) or an equal volume of saline at 113 and 114 days gestation. Fetal blood samples were collected throughout the experimental period to monitor fetal wellbeing. Animals were euthanized (sodium pentobarbitone; Lethabarb, Virbac, Australia) at either 115 days (24 h following the second betamethasone administration) or 125 days (11 days following the second dose of betamethasone). Fetal brains were removed, weighed and the two hemispheres cut sagitally before the right hemisphere was fixed in 4% paraformaldehyde and later processed for histological analysis and pieces of the left hemisphere snap-frozen and stored at −70°C for future studies.

Fetal brain sections were analyzed in duplicate using glial fibrillary acidic protein (GFAP; mouse anti-rabbit; diluted 1:400; Sigma–Aldrich, St. Louis, MO, USA), 2′,3′-cyclic-nucleotide 3′-phosphodiesterase (CNPase; mouse anti-human; diluted 1:300; Sigma–Aldrich, St. Louis, MO, USA), oligodendrocyte transcription factor 2 (Olig2; mouse anti-human; diluted 1:1,000; MerckMillipore, Australia) and myelin basic protein (MBP; rat monoclonal; diluted 1:100; MerckMillipore, Australia). Briefly, sections were dewaxed and rehydrated before antigen retrieval was carried out in citric acid buffer (0.1 M; Sigma-Aldrich, Australia). The sections were incubated overnight at 4°C with primary antibody followed by secondary antibody (MBP goat anti-rat; diluted 1;500; Vector Laboratories, Burlingame, CA, USA; GFAP, CNPase, Olig2 goat anti-mouse; diluted 1:200; Vector Laboratories, Burlingame, CA, USA) and streptavidin horseradish peroxidase (diluted 1:200; GE Healthcare, USA) before being visualized with diaminobenzidine (DAB; Thermo Fisher Scientific, Waltham, MA, USA). Negative controls that omitted the primary antibody were included in each run. The sections were viewed at 400× magnification (Olympus BX-41, Japan) with three fields of view used for each region from each section. Images were analyzed using ImageJ software (National Institutes of Health, USA).

Data are expressed as mean ± standard error of the mean (SEM) and analyzed by three-way analysis of variance (ANOVA) with fetal growth (control or FGR), maternal glucocorticoid treatment (vehicle or BM) and age (115 days or 125 days) as fixed variables (GraphPad Prism 8, GraphPad Software Inc., La Jolla, CA, USA). Fetal blood parameters were analyzed over time using a four-way repeated measures ANOVA (SigmaStat 12, Systat Software, USA). Where significant interactions were observed, differences between groups were isolated using a one-way ANOVA or mixed model with Tukey’s multiple comparison test performed as required. Statistical significance was accepted when *P* < 0.05.

## Results

Fetal arterial blood samples were taken over the experimental period to monitor fetal wellbeing (Table 1). Baseline values were combined for all control and all FGR animals. The FGR groups (FGR and FGR+BM) were hypoxic compared to control animals (control and control+BM) at baseline recording (mean PaO_2_ 19.6 ± 0.8 mmHg vs. 23.0 ± 0.6 mmHg; *P* < 0.001). Following betamethasone, both partial pressure of oxygen (PaO_2_) and oxygen saturation (SaO_2_) were significantly reduced in the 115 days FGR+BM fetuses. There were no significant differences in oxygen levels in any other group (Table 1). FGR fetuses were hypoglycaemic at baseline when compared to control fetuses. Following each betamethasone administration, both control and FGR fetuses in both the 115 days and 125 days cohort had significantly higher glucose levels. This persisted at post mortem in the 115 days fetuses but had returned to baseline values by post mortem in the 125 days fetuses. Fetal lactate levels were also significantly increased in all fetuses exposed to betamethasone. FGR+BM fetuses had increased lactate levels at post mortem in the 115 days cohort (Table 1).

**Table 1:** Fetal characteristics and arterial blood parameters.

		Baseline		115 days				125 days			
		Control	FGR	Control	FGR	Control+BM	FGR+BM	Control	FGR	Control+BM	FGR+BM
Number (M/F)				9 (5/4)	9 (5/4)	8 (6/2)	8 (6/2)	9 (3/6)	7 (4/3)c	5 (2/3)	5 (1/4)
Gestational age at post mortem (d)				114.8 ± 0.7	114.8 ± 0.7	115.1 ± 0.8	115.1 ± 0.8	125.4 ± 0.5	125.9 ± 0.6	123.4 ± 0.2	124 ± 0.0
PaO_2_ (mmHg)	Baseline	23.0 ± 0.6	**19.6 ± 0.8^#^**								
	BM1 +6 h			23.4 ± 0.9	20.5 ± 1.3	23.8 ± 1.2	20.8 ± 0.8	22.8 ± 1.1	21.5 ± 2.2	23.7 ± 0.7	20.2 ± 2.0
	BM2 +6 h			23.8 ± 1.2	22.1 ± 1.6	23.6 ± 1.9	**18.9 ± 1.2^#^**	23.3 ± 2.0	21.9 ± 2.9	23.6 ± 0.8	19.7 ± 0.9
	PM			21.0 ± 1.4	17.5 ± 1.6	23.7 ± 1.3	**16.4 ± 1.6^#^**	21.8 ± 2.5	19.2 ± 2.5	28.6 ± 0.0	17.3 ± 1.8
SaO_2_ (%)	Baseline	63.6 ± 1.5	**52.4 ± 2.7^#^**
	BM1 +6 h			61.7 ± 2.5	55.3 ± 3.4	61.7 ± 3.3	52.3 ± 2.2	63.7 ± 3.4	61.5 ± 6.8	70.5 ± 2.5	54.7 ± 6.3
	BM2 +6 h			63.0 ± 2.1	57.0 ± 4.1	61.3 ± 6.5	**46.3 ± 4.7^#^**	64.2 ± 7.3	58.1 ± 7.8	67.6 ± 1.1	52.3 ± 2.8
	PM			55.8 ± 4.3	47.6 ± 3.2	65.2 ± 3.5	36.3 ± 6.6^*#^	65.2 ± 4.4	50.3 ± 8.9	76.6 ± 0.0	45.2 ± 5.2
Glucose (mmol/l)	Baseline	0.7 ± 0.0	**0.6 ± 0.0^#^**
	BM1 +6 h			0.8 ± 0.1	0.7 ± 0.1	1.9 ± 0.2^*#^	1.4 ± 0.1^*#^	0.9 ± 0.1	0.7 ± 0.1	1.8 ± 0.2^*#^	1.7 ± 0.2^*#^
	BM2 +6 h			0.8 ± 0.1	0.7 ± 0.1	2.4 ± 0.2^*#^	1.6 ± 0.2^*#^	0.8 ± 0.1	0.6 ± 0.1	1.9 ± 0.3^*#^	2.1 ± 0.3^*#^
	PM			0.6 ± 0.1	0.5 ± 0.1	1.6 ± 0.1^*#^	1.0 ± 0.1^*#^	0.8 ± 0.1	0.8 ± 0.2	0.9 ± 0.0	0.6 ± 0.1
Lactate (mmol/l)	Baseline	1.1 ± 0.0	1.2 ± 0.0
	BM1 +6 h			1.1 ± 0.1	1.4 ± 0.1	2.6 ± 0.2^*#^	2.5 ± 0.2^*#^	1.4 ± 0.1*	1.5 ± 0.1	2.4 ± 0.3^*#^	2.5 ± 0.2^*#^
	BM2 +6 h			1.3 ± 0.1	1.5 ± 0.2	2.5 ± 0.1^*#^	2.6 ± 0.2^*#^	1.5 ± 0.2*	1.7 ± 0.2	2.8 ± 0.6^*#^	2.4 ± 0.3^*#^
	PM			1.2 ± 0.1	1.7 ± 0.3	2.2 ± 0.3^*^	3.5 ± 0.6^*#^	1.6 ± 0.1*	3.1 ± 1.0^*^	0.6 ± 0.0	1.6 ± 0.1

Data are presented as mean ± SEM. Bold values indicate significant differences. ^*^P < 0.05, compared with baseline within the group. ^#^*P* < 0.05, compared to same-age control at the same time.

**Figure 1:**
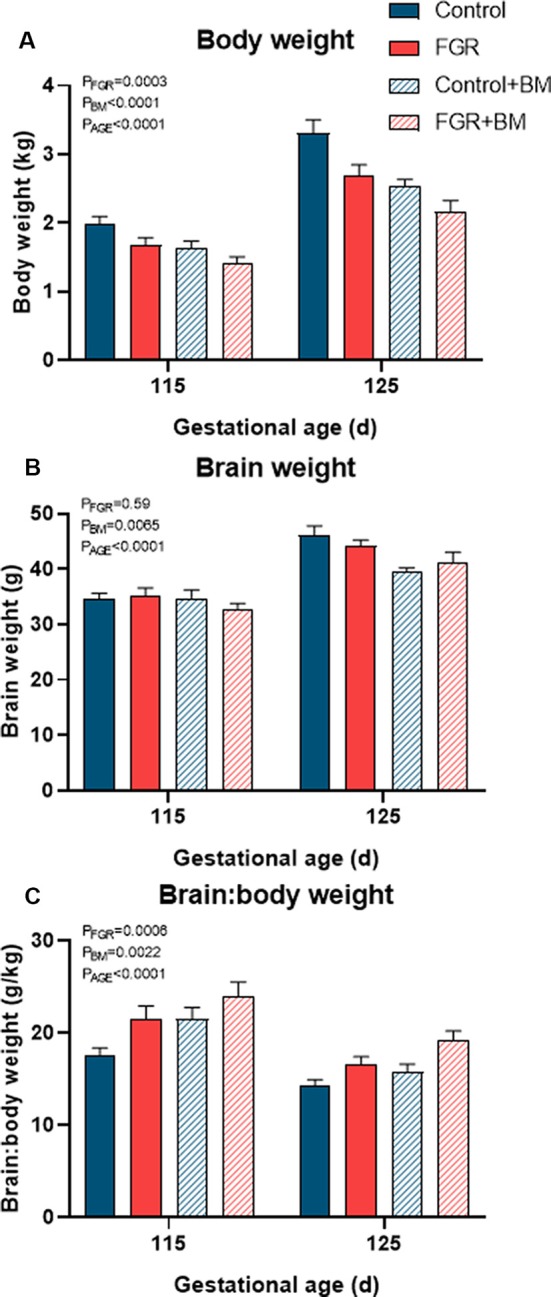
Fetal weights at post mortem. Fetal body weight **(A)**, brain weight **(B)** and the brain: body weight ratio **(C)** at post mortem. Data are presented as mean ± SEM.

Fetal weights at the time of post mortem are shown in Figure 1. FGR fetuses weighed significantly less than controls (*P* < 0.001) while betamethasone treatment also significantly reduced fetal body weight (*P* < 0.001). FGR did not affect fetal brain weight (*P* = 0.59), but the brain:body weight ratios were increased in all FGR fetuses compared to controls (*P* < 0.001), indicative of brain sparing. Betamethasone reduced fetal brain weight (*P* = 0.007) and increased brain:body weight ratios (*P* = 0.002). Fetal age (115 days vs. 125 days) had a significant effect on weight parameters (Figure 1).

**Figure 2:**
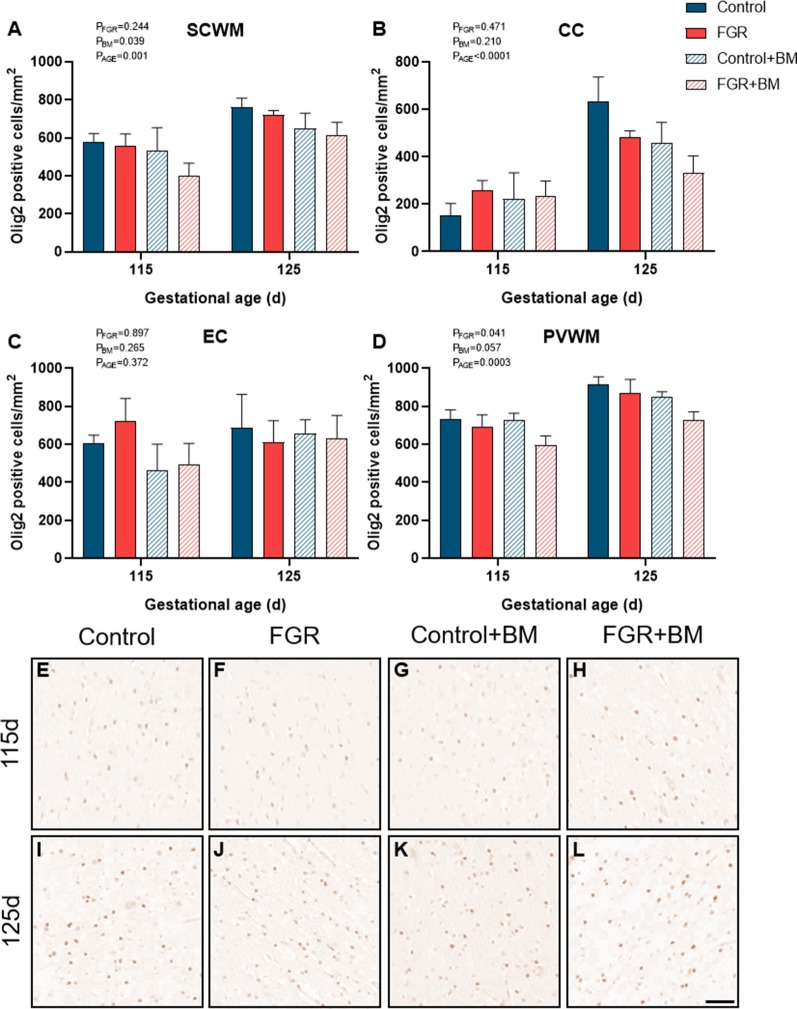
Olig2 immunoreactivity. The number of Olig2 positive cells in the subcortical white matter (SCWM; **A**), corpus callosum (CC; **B**), external capsule (EC; **C**) and periventricular white matter (PVWM; **D**) and photomicrographs of the SCWM from 115 days control **(E)**, fetal growth restriction (FGR; **F**), control+BM **(G)**, FGR+BM **(H)** and 125 days control **(I)**, FGR **(J)**, control+BM **(K)** and FGR+BM **(L)** fetuses. Data are presented as mean ± SEM, scale bar = 50 μm.

Within the fetal brain, the number of Olig2 positive oligodendrocyte lineage cells (Figure 2) was significantly reduced in FGR fetuses when compared to control in the periventricular white matter (PVWM; *P* = 0.04). Betamethasone treatment caused a significant decrease in oligodendrocyte lineage cells within the subcortical white matter (SCWM; *P* = 0.04). Oligodendrocyte cell counts were significantly increased in 125 days brains compared to 115 days brains in the SCWM (*P* = 0.001), corpus callosum (CC; *P* < 0.001) and PVWM (*P* < 0.001).

**Figure 3:**
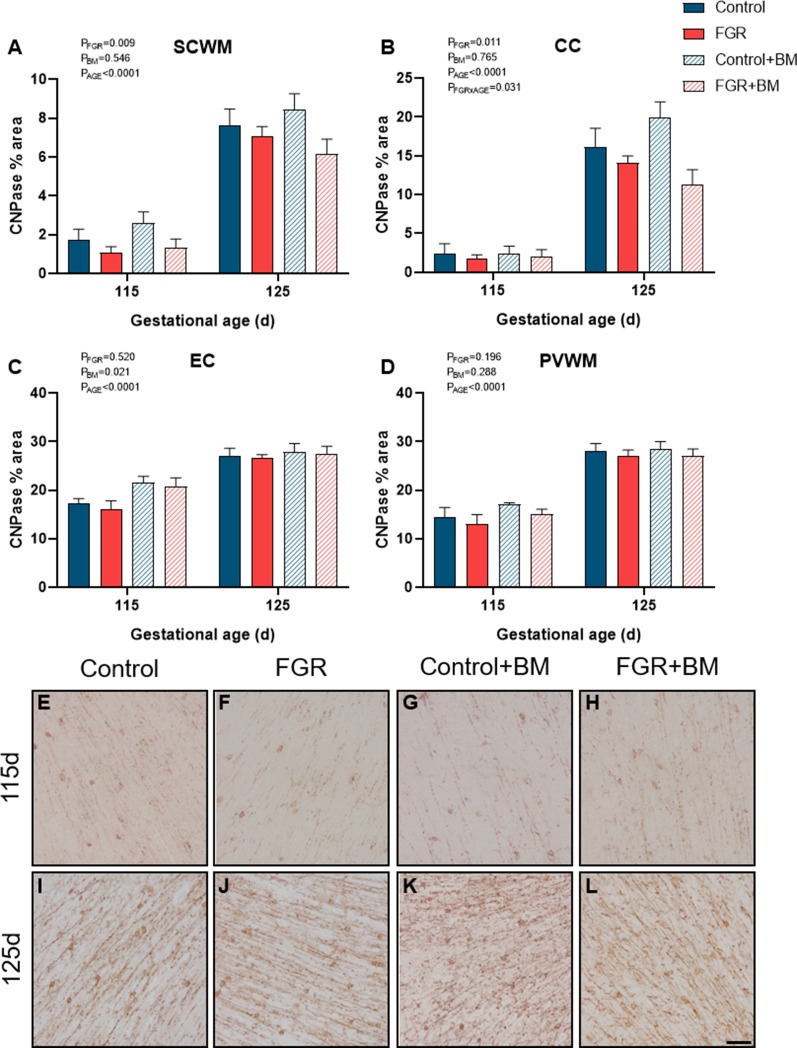
CNPase immunoreactivity. The % area of CNPase positive staining in the SCWM **(A)**, CC **(B)**, EC **(C)** and PVWM **(D)** and photomicrographs of the SCWM from 115 days control **(E)**, FGR **(F)**, control+BM **(G)**, FGR+BM **(H)** and 125 days control **(I)**, FGR **(J)**, control+BM **(K)** and FGR+BM **(L)** fetuses. Data are presented as mean ± SEM, scale bar = 50 μm.

**Figure 4:**
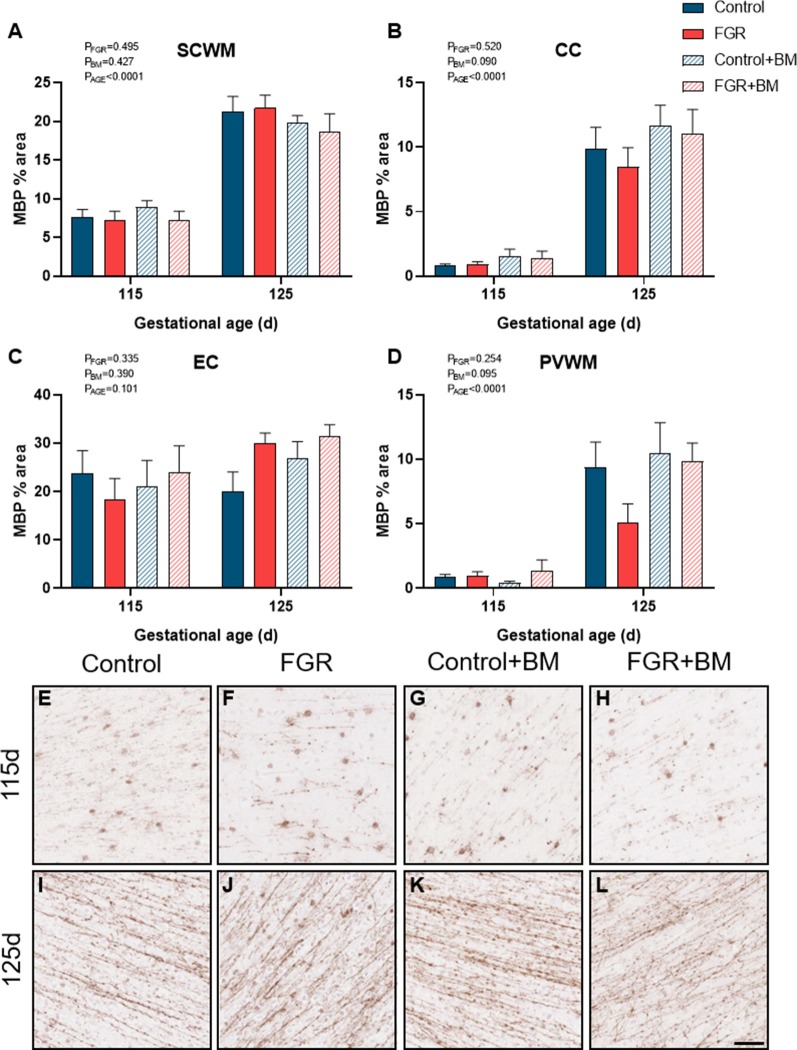
MBP immunoreactivity. The % area of MBP positive staining in the SCWM **(A)**, CC **(B)**, EC **(C)** and PVWM **(D)** and photomicrographs of the SCWM from 115 days control **(E)**, FGR **(F)**, control+BM **(G)**, FGR+BM **(H)** and 125 days control **(I)**, FGR **(J)**, control+BM **(K)** and FGR+BM **(L)** fetuses. Data are presented as mean ± SEM, scale bar = 50 μm.

We utilized two staining markers of myelination density within the developing brain, CNPase, and MBP. There was significantly less CNPase-positive myelin in FGR fetuses compared to control within the SCWM (*P* = 0.009) and CC (*P* = 0.01; Figure 3). Betamethasone administration significantly increased CNPase-positive myelin staining in the external capsule (EC; *P* = 0.02) but did not alter staining in any other region examined. Overall, MBP density was not changed by FGR or betamethasone administration (Figure 4). Over the 10 days of study, the degree of myelination was significantly increased from 115 days to 125 days gestation, as evidenced by increased density of staining for both CNPase and MBP (Figures 3, 4). Interestingly, we observed that MBP-positive myelin density was increased >10-fold in the PVWM of control brains from 115 days to 125 days gestation, consistent with this period being critical for myelin production, however, within the FGR brains MBP-positive myelin density was increased ~5-fold within the PVWM over the same period. Both CNPase and MBP staining appeared to be disorganized in FGR brains, with interrupted tracts present within the white matter.

**Figure 5:**
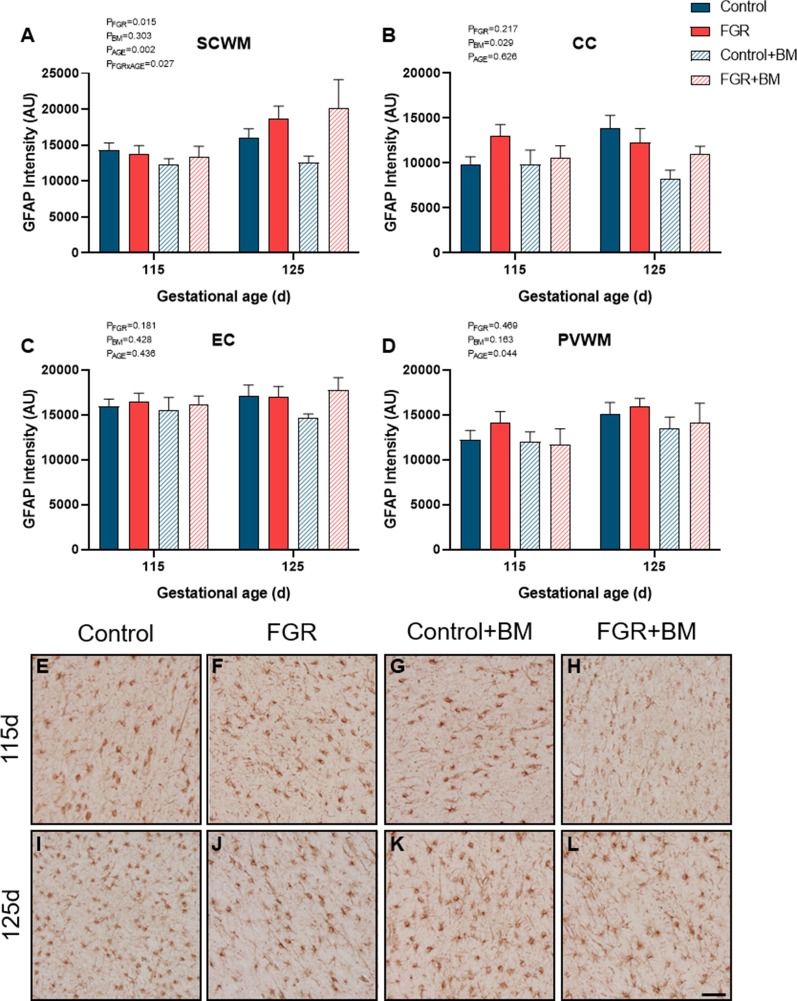
GFAP immunoreactivity. The intensity of GFAP positive staining in the SCWM **(A)**, CC **(B)**, EC **(C)** and PVWM **(D)** and photomicrographs of the SCWM from 115 days control **(E)**, FGR **(F)**, control+BM **(G)**, FGR+BM **(H)** and 125 days control **(I)**, FGR **(J)**, control+BM **(K)** and FGR+BM **(L)** fetuses. Data are presented as mean ± SEM, scale bar = 50 μm.

We assessed GFAP immunoreactivity as a measure of astrogliosis within the fetal brain (Figure 5). Astrogliosis was evident within the SCWM in FGR brains (*P* = 0.02). Conversely, astrocyte staining intensity was significantly reduced following betamethasone administration within the CC (*P* = 0.03), contributed by a significant reduction in astrocytic staining within control brains between the control and control+BM 125 days cohort (*P* = 0.04).

## Discussion

We set out to examine whether a single course of antenatal betamethasone altered white matter brain development in fetal sheep that were either appropriately grown or compromised by FGR. We administered a course of 2 maternal injections of betamethasone separated by 24 h and assessed fetal cerebral white matter at 48 h post initial exposure to antenatal glucocorticoids (115 days gestation), or 10 days later (125 days gestation) in a separate cohort. Our results confirm that placental insufficiency and subsequent FGR adversely affects white matter brain development and, for the first time, we show that antenatal betamethasone does not exacerbate white matter deficits in FGR offspring. That is, a single course of betamethasone caused detrimental effects on fetal growth and brain development, but these were similar in appropriately grown and FGR fetuses. Within the developing brain, betamethasone exposure significantly reduced the number of oligodendrocyte lineage cells but promoted CNPase-positive myelin density in an area-specific manner within the cerebral white matter. Betamethasone demonstrated anti-inflammatory effects within control brains only, as evidenced by decreased astrocyte density within the CC. Reassuringly, our results do not support our hypothesis that antenatal glucocorticoids worsen white matter injury in FGR fetuses, but rather demonstrate that neuropathology associated with FGR is present antenatally and is principally caused by placental insufficiency.

A clinical course of antenatal betamethasone significantly reduced body weight in both appropriately grown and FGR fetuses (Figure 1). Unsurprisingly, the most severe growth restriction was observed in the FGR+BM cohort, weighing 35% less than control fetuses at 125 days gestation. Single and repeat courses of antenatal glucocorticoids reduce body weight in fetal sheep (Sloboda et al., 2000; Miller et al., 2007, 2012), with a dose-dependent effect (Ikegami et al., 1997). A large population-based Finnish study confirms that birth weight is significantly reduced in infants exposed to antenatal steroids in infants born preterm, near-term or at term (Rodriguez et al., 2019). This study did not stratify for infants with FGR, but noted that 44% of their glucocorticoid-exposed infants were born at term and therefore unnecessarily received steroids. A dose-dependent relationship on fetal growth is also present in human infants exposed to single and repeat courses of antenatal steroids (Murphy et al., 2012).

FGR was induced *via* a model of placental insufficiency that produced fetal hypoxia and hypoglycemia and resulted in reduced body weight of 15.6% at 115 days and 18% at 125 days compared to appropriately grown controls, and with apparent brain sparing. Despite brain sparing, white matter pathology was observed, with a decrease in total oligodendrocytes within the PVWM, and reduced myelin density (CNPase-positive) within the subcortical white matter and CC. The decrease in the oligodendrocyte pool within the PVWM is important, as injury to this brain region is highly associated with neurodevelopmental deficits (Volpe, 2009), and is a common imaging abnormality in growth-restricted infants (Padilla-Gomes et al., 2007). We observed sparse MBP-positive mature myelin staining at 115 days in the fetal sheep brain, but this was greatly increased by 125 days, confirming previous findings (Back et al., 2006; Antonow-Schlorke et al., 2009). Importantly, while control brains demonstrated a >10-fold increase in MBP-positive myelin density over this period, the FGR brains showed only a 5-fold increase in myelination, potentially contributed by the decrease observed in the oligodendrocyte pool. This significant myelination period between 115 days and 125 days gestation in the fetal sheep brain is approximately equivalent to white matter development in the human brain between 30–36 weeks (Back et al., 2006; Alves de Alencar Rocha et al., 2017), and this also corresponds to a high-risk period for preterm birth (and steroid exposure) in FGR infants (Lees et al., 2013). Our results also confirm that there are significant regional differences in the maturation of cerebral white matter and that third-trimester placental insufficiency adversely impacts white matter development. Reassuringly we did not find that antenatal glucocorticoids exacerbated white matter pathology in the FGR group, however, betamethasone did independently impact brain development in both appropriately grown controls and FGR fetuses.

The administration of betamethasone significantly reduced the number of oligodendrocyte lineage cells within the subcortical white matter in both control and FGR brains, with the lowest number of oligodendrocytes observed in the FGR+BM cohort. We did not observe any change to MBP-positive myelination with betamethasone exposure at either 115 days or 125 days. This is surprising in light of findings by Antonow-Schlorke and colleagues (Antonow-Schlorke et al., 2009) who demonstrated acute deficits in MBP-positive myelination with a single dose of betamethasone in sheep, but also showed no lasting deficits unless repeat steroids were administered. Similarly, a study in baboons shows that repeat courses of steroids have more profound adverse effects on the cerebral white matter than a single course (Shields et al., 2012). An unexpected finding was that betamethasone exposure induced a significant overall increase in CNPase-positive myelin density within the external capsule, and sub-analysis shows that this was contributed by an acute increase in myelination in the 115 days group (control and FGR). Glucocorticoids are potent mediators of cell maturation and differentiation, hence the use of synthetic glucocorticoids for fetal lung maturation before preterm birth (Bolt et al., 2001). Therefore it is conceivable that betamethasone might accelerate the process of myelination within the brain, and indeed, others have shown this to be the case (Raschke et al., 2008). This was, however, a transitory observation in the current study with no difference seen at 125 days gestation. Further, a noted benefit and mode of action of synthetic glucocorticoids is *via* anti-inflammatory effects within the immature lung (Bolt et al., 2001). In the current study, we also found that betamethasone demonstrated anti-inflammatory effects, although within control brains only, as evidenced by decreased astrocyte density within the CC. This result is in keeping with a previous study in guinea pigs showing that antenatal betamethasone decreased the density of astrocytes within the brain of appropriately grown, but not FGR, fetuses, with this effect only observed in males (McKendry et al., 2010). We did not have adequate numbers of males and females to undertake separate analysis of sex differences in the current study, but we have previously published data to show that antenatal betamethasone has differential effects on fetal growth and metabolism in males and females (Miller et al., 2012), and therefore further studies are encouraged to examine sex-specific effects. Taken together, the results obtained in this study, and other preclinical studies to date, strongly suggest that antenatal glucocorticoids have subtle but important modulatory effects on cerebral white matter maturation that are region specific and appear dependent on the timing of steroid exposure, and the use of single or repeat doses (Antonow-Schlorke et al., 2009; Shields et al., 2012). It is not yet established whether myelination deficits are transient (delayed) or persist after birth. We did not set out to specifically examine mechanisms of glucocorticoid-induced alterations to white matter development, however, our results suggest that glucocorticoids alter oligodendrocyte maturation rather than directly affecting the myelin sheath.

Synthetic glucocorticoids are widely used during pregnancy but our results suggest that the actions of glucocorticoids are not specific to the immature lung, also mediating brain development. There is emerging clinical follow-up data to support this finding. The use of antenatal glucocorticoids is linked to a significant increase in psychiatric disorders in school-age children that appears to persist in adolescence (Khalife et al., 2013). Furthermore, this effect can be isolated to glucocorticoids rather than preterm birth, since in a population of adolescents who were exposed to a single course of antenatal glucocorticoids but subsequently delivered at term, it was found that cognitive and behavioral control were significantly reduced (Ilg et al., 2018).

In the present study, we focused on cerebral white matter development, however, both FGR and synthetic glucocorticoids have other established actions on the immature brain. Of particular importance are potential effects on the cerebrovasculature given the strong vasoactive actions of glucocorticoids and the vulnerability of the neurovascular unit components in FGR lambs (Castillo-Melendez et al., 2015). Antenatal betamethasone has distinctly different actions on cardiac output and cerebral blood flow redistribution in appropriately grown and FGR humans and sheep (Edwards et al., 2002; Miller et al., 2009), with FGR fetuses responding to betamethasone with significant vasodilation. A single course of antenatal steroids is also shown to induce cortical hyperactivity and markedly alters sleep architecture in appropriately grown fetal sheep (Davidson et al., 2011) but effects on growth-restricted fetuses have not yet been examined. In the current study, all fetuses remained *in utero* which is likely to be a relatively protective environment for brain development, and therefore future studies should re-create the clinical scenario in which many infants would be born preterm, soon after steroid exposure. Preterm birth and ventilation are linked with neuropathology in both appropriately grown and FGR infants (Barton et al., 2015; Malhotra et al., 2018).

Using two myelin protein markers at two timepoints in late gestation also provides us with information about the normal spatiotemporal profile of myelin production. CNPase accounts for approximately 5% of total myelin protein in the adult brain, whereas MBP is quantitatively greater than CNPase comprising ~30% of total myelin (Trapp et al., 1988; Baumann and Pham-Dinh, 2001). Our results show regional differences in the developmental profile of myelin production. The external capsule was already well myelinated at 115 days gestation for both CNPase and MBP protein, and showed the least change over the following 10 days, suggesting relative maturity of this area. In contrast, very low levels of myelination were observed in the CC at 115 days using both markers, and both were increased to a similar degree in control brains at 125 days, but not in the FGR brains. In the subcortical white matter, MBP was more strongly evident than CNPase at an earlier age, with both proteins significantly upregulated between 115 and 125 days. Whereas in the PVWM the CNPase protein was present earlier and MBP was virtually absent, with MBP density showing a 10-fold increase over the 10 days. Myelination of the fetal brain occurs earlier and more rapidly in the fetal sheep brain than the human (Back et al., 2012), but these data confirm that antenatal compromise (e.g., FGR) or exposure to exogenous glucocorticoids during rapid myelination has region-specific adverse effects.

Here, we have shown that placental insufficiency and FGR disrupt cerebral white matter development that can already be detected at a time point in pregnancy when antenatal steroids might commonly be administered (115 days in fetal sheep, equivalent to ~30 weeks of human gestation). In the absence of antenatal glucocorticoid exposure, FGR is associated with postnatal deficits in motor function, cognition, and behavior that are underpinned by altered brain structure (Miller et al., 2016). Betamethasone administration resulted in independent subtle adverse effects on white matter brain development in appropriately grown and FGR fetuses, but antenatal glucocorticoids did not exacerbate white matter pathology. This data shows that the administration of antenatal glucocorticoids should be targeted to the pregnancies of greatest risk for preterm birth because of the effects of glucocorticoids on the developing brain.

## Data Availability Statement

The datasets generated for this study are available on request to the corresponding author.

## Ethics Statement

The animal study was reviewed and approved by Monash Medical Centre, Animal Ethics Committee A.

## Author Contributions

TY, MC-M, BA, GP, GJ, and SM contributed to the conception and design of the study. AS, TY, MC-M, BA, AM, GP, LC, GJ, and SM contributed to the acquisition, analysis, and interpretation of the data. AS and SM drafted the initial manuscript and all authors edited the manuscript for intellectual content and approved the final version for submission.

## Conflict of Interest

The authors declare that the research was conducted in the absence of any commercial or financial relationships that could be construed as a potential conflict of interest.
